# Indications and hemoglobin thresholds for red blood cell transfusion and iron replacement in adults with gastrointestinal bleeding: An algorithm proposed by gastroenterologists and patient blood management experts

**DOI:** 10.3389/fmed.2022.903739

**Published:** 2022-09-15

**Authors:** Miguel Montoro, Mercedes Cucala, Ángel Lanas, Cándido Villanueva, Antonio José Hervás, Javier Alcedo, Javier P. Gisbert, Ángeles P. Aisa, Luis Bujanda, Xavier Calvet, Fermín Mearin, Óscar Murcia, Pilar Canelles, Santiago García López, Carlos Martín de Argila, Montserrat Planella, Manuel Quintana, Carlos Jericó, José Antonio García Erce

**Affiliations:** ^1^Unidad de Gastroenterología, Hepatología y Nutrición, Hospital Universitario San Jorge, Huesca, Spain; ^2^Departamento de Medicina, Universidad de Zaragoza, Zaragoza, Spain; ^3^Instituto Aragonés de Ciencias de la Salud (IACS), Zaragoza, Spain; ^4^Instituto de Investigación Sanitaria Aragón (IIS), Zaragoza, Spain; ^5^Vifor Pharma, Barcelona, Spain; ^6^Servicio de Aparato Digestivo, Hospital Clínico Universitario “Lozano Blesa”, Zaragoza, Spain; ^7^Centro de Investigación Biomédica en Red de Enfermedades Hepáticas y Digestivas (CIBEREHD), Madrid, Spain; ^8^Servei de Digestiu, Hospital de la Santa Creu y Sant Pau, Universidad Autónoma de Barcelona, Barcelona, Spain; ^9^Unidad de Gestión Clínica de Aparato Digestivo, Hospital Universitario Reina Sofía de Córdoba, Córdoba, Spain; ^10^Servicio de Aparato Digestivo, Hospital Universitario Miguel Servet, Zaragoza, Spain; ^11^Servicio de Aparato Digestivo, Hospital Universitario de La Princesa, Madrid, Spain; ^12^Instituto de Investigación Sanitaria Princesa (IIS-IP), Universidad Autónoma de Madrid (UAM), Madrid, Spain; ^13^Servicio de Aparato Digestivo, Hospital Universitario Costa del Sol, Marbella, Spain; ^14^Servicio de Aparato Digestivo, Hospital Universitario Donostia, Donostia, Spain; ^15^Instituto de Investigación Sanitaria Biodonostia, Universidad del País Vasco (UPV/EHU), Donostia, Spain; ^16^Servei de Digestiu, Corporació Sanitaria Park Taulí, Sabadell, Spain; ^17^Department of Medicine, Universidad Autónoma de Barcelona, Barcelona, Spain; ^18^Servicio de Aparato Digestivo, Centro Médico Teknon, Barcelona, Spain; ^19^Servicio de Aparato Digestivo, Hospital General Universitario de Alicante, Alicante, Spain; ^20^Servicio de Aparato Digestivo, Hospital General Universitario de Valencia, Valencia, Spain; ^21^Servicio de Aparato Digestivo, Hospital Universitario Ramón y Cajal, Madrid, Spain; ^22^Servei de Digestiu, Hospital Universitario Arnau de Vilanova, Lleida, Spain; ^23^Department of Medicine, Universidad de Lleida, Lleida, Spain; ^24^Servicio a Medicina Intensiva, Hospital Universitario La Paz (IdiPAZ), Madrid, Spain; ^25^PBM Group, Hospital La Paz Institute for Health Research (IdiPAZ), Madrid, Spain; ^26^Servicio de Medicina Interna, Complex Hospitalari Moisès Broggi, Sant Joan Despí, Barcelona, Spain; ^27^Grupo Español de Rehabilitación Multimodal (GERM), Zaragoza, Spain; ^28^Banco de Sangre y Tejidos de Navarra, Servicio Navarro de Salud, Osasunbidea, Pamplona, Spain; ^29^Instituto Aragonés de Ciencias de la Salud, Universidad de Zaragoza, Zaragoza, Spain

**Keywords:** anemia, ferric carboxymaltose (FCM), gastrointestinal bleeding, iron supplementation, patient blood management, transfusion

## Abstract

Gastrointestinal (GI) bleeding is associated with considerable morbidity and mortality. Red blood cell (RBC) transfusion has long been the cornerstone of treatment for anemia due to GI bleeding. However, blood is not devoid of potential adverse effects, and it is also a precious resource, with limited supplies in blood banks. Nowadays, all patients should benefit from a patient blood management (PBM) program that aims to minimize blood loss, optimize hematopoiesis (mainly by using iron replacement therapy), maximize tolerance of anemia, and avoid unnecessary transfusions. Integration of PBM into healthcare management reduces patient mortality and morbidity and supports a restrictive RBC transfusion approach by reducing transfusion rates. The European Commission has outlined strategies to support hospitals with the implementation of PBM, but it is vital that these initiatives are translated into clinical practice. To help optimize management of anemia and iron deficiency in adults with acute or chronic GI bleeding, we developed a protocol under the auspices of the Spanish Association of Gastroenterology, in collaboration with healthcare professionals from 16 hospitals across Spain, including expert advice from different specialties involved in PBM strategies, such as internal medicine physicians, intensive care specialists, and hematologists. Recommendations include how to identify patients who have anemia (or iron deficiency) requiring oral/intravenous iron replacement therapy and/or RBC transfusion (using a restrictive approach to transfusion), and transfusing RBC units 1 unit at a time, with assessment of patients after each given unit (i.e., “don’t give two without review”). The advantages and limitations of oral versus intravenous iron and guidance on the safe and effective use of intravenous iron are also described. Implementation of a PBM strategy and clinical decision-making support, including early treatment of anemia with iron supplementation in patients with GI bleeding, may improve patient outcomes and lower hospital costs.

## Introduction

Acute or chronic gastrointestinal (GI) bleeding affects 47 in 100,000 people worldwide (2) and is one of the most significant clinical problems observed by gastroenterologists, hepatologists, internal medicine physicians, and surgeons (3–6). Upper GI bleeding is associated with mortality rates of 3–14% (4), while a mortality rate of approximately 3% has been reported in patients with lower GI bleeding (7). However, rapid correction of anemia and hypotension may reduce bleeding-associated mortality by preventing cardiovascular decompensation (8–10).

Red blood cell (RBC) transfusion has long been the cornerstone of treatment for anemia due to GI bleeding although the rate of transfusions vary, with 6% of RBC transfusions in Northern Spain (11) and 11–14% of RBC transfusions in England (12, 13) being given due to GI bleeding. However, evidence is accumulating of a dose-response relationship between transfusion and increased patient morbidity, mortality, and length of hospital stay (14–18). Therefore, there has recently been a shift toward a more restrictive use of RBC transfusion on the basis that outcomes are similar or better than when a more liberal strategy is used (19–29). Reduced rates of rebleeding (21, 22), reduced transfusion-associated risks (30, 31), lower all-cause mortality (21), and shorter hospital stays (22) have all been reported when a restrictive rather than liberal transfusion strategy had been implemented. Furthermore, no differences in the risk of ischemic events (21) or major adverse cardiovascular events (25) have been noted between liberal and restrictive strategies. A restrictive transfusion strategy can also reduce healthcare resource utilization and costs (32–34). For example, the United Kingdom’s National Health Service (NHS) saved £3.3 million in the year following the introduction of a restrictive transfusion policy for lower GI bleeding (34). Cochrane meta-analyses support the use of restrictive RBC transfusion strategies across a broad range of clinical indications, including in hemodynamically stable patients with GI bleeding (35). A restrictive approach is now commonly used, resulting in a global reduction in RBC transfusions (36) and transfusion-associated risks. Importantly, reduced demand for blood supplies helps to ensure that blood is available for those who need it most, which is important given that currently approximately 25 million units of blood are transfused to more than 5 million patients each year in Europe (37). With an aging population and a decreasing number of blood donors, it is expected that more and more countries will experience challenges in ensuring that blood supplies are adequate (38). Indeed, blood shortages have been reported in several European countries during health emergencies in recent years (39). Despite the continued efforts of the European Commission to ensure the optimum use of blood components (40), data from the NHS suggest that approximately 15–20% of RBC transfusions are used inappropriately (41). Moreover, the persistent variation in blood utilization across European Union member states indicates that the inappropriate use of blood supplies is widespread (42). Despite the benefits of a restrictive approach to transfusion, consensus recommendations are lacking, and major shortcomings have been identified in many clinical practice guidelines for transfusion practice (43). Applying consistent criteria to clinical decision-making regarding the eligibility and timing of transfusion in patients with GI bleeding is crucial (30).

Patient blood management (PBM) is an evidence-based bundle of care that aims to optimize outcomes for all patients with bleeding potential by managing and preserving blood. The concept of PBM is built on 3 pillars: (1) optimization of RBC mass, including the use of iron replacement therapy and/or erythropoiesis-stimulating agents where needed; (2) minimization of blood loss/bleeding; and (3) optimization of the patient’s tolerance of anemia (36, 44, 45). PBM can reduce patient mortality and morbidity (46) and its incorporation into healthcare management has the potential to bring benefits to many patients and healthcare institutions (47–50). It is also crucial to raise awareness about the importance of preserving and managing the patient’s own blood and maintaining well-functioning bone marrow erythropoiesis, rather than routinely resorting to the use of donor blood. This approach should improve patient outcomes as well as reduce the rate of over-transfusion, thereby preventing many transfusions that would otherwise have been deemed appropriate.

We therefore developed the current protocol, based on the three pillars of PBM, with the aim of optimizing the management of patients with anemia and iron deficiency due to GI bleeding in clinical practice. For this purpose, it was deemed very important to include the expert opinion of PBM experts, because of their high awareness of the need to save blood and minimize healthcare costs.

## Aims of the protocol

This protocol was commissioned and approved by the Spanish Association of Gastroenterology (Asociación Española de Gastroenterología [AEG]), after an expert review was performed by professionals from 16 Spanish hospitals, who were also part of the Working Group on “Esophagus, Stomach, and Duodenum” for the AEG. The review was conducted in collaboration with internal medicine physicians, intensive care specialists, and hematologists with advanced knowledge of PBM programs. This was not a formal systematic literature review but was based on a review of the literature to provide best practice advice statements. No formal rating of the quality of evidence or strength of recommendation was performed.

All the authors were invited for their experience, prestige, academic recognition, and representation in their respective societies. Most of the authors worked on the initial reference document, which was presented at a Congress of the AEG. Subsequently, it was disseminated among the members of the AEG and underwent internal and external peer-review through the standard procedures of Clinical Gastroenterology and Hepatology. Several years later, the document was updated with the comments and criticism received and presented for additional rounds of review and discussion; it was unanimously approved 6 months later. A shorter online version of the protocol is also available on the AEG website: https://www.aegastro.es/documents/prodiggest/Prodiggest-Management-of-anaemia-and-iron-deficiency-in-gastrointestinal-bleeding.pdf.

The primary goal of the project was to develop a healthcare protocol for the management of anemia and iron deficiency associated with GI bleeding. Therefore, current indications for RBC transfusion were reviewed in the context of patients with GI bleeding. The protocol aimed to provide guidance on achieving an adequate balance between the use and overuse of RBC transfusion according to a restrictive model, promoting the use of restrictive rather than liberal criteria and a 1 blood unit policy (i.e., *“don’t give two without review”*). Other aims of the protocol were to provide information on the advantages and limitations of oral versus intravenous iron in patients with GI bleeding and to provide guidance on the safe and effective use of intravenous iron for the treatment of blood loss associated with GI bleeding.

This protocol focuses on the management of acute anemia or iron deficiency in patients with acute GI bleeding (with or without portal hypertension) and on the management of chronic anemia or iron deficiency due to fecal occult blood loss. The protocol is not intended for use in pediatric patients or for patients with anemia or iron deficiency due to causes other than GI bleeding such as inflammatory bowel disease. The management of other aspects of GI bleeding are also out of the scope of the protocol (e.g., evaluating the extent of blood loss, resuscitation measures, identifying and treating the sources of bleeding).

This protocol is relevant to clinicians and nurses who treat adults with acute or chronic GI bleeding, and it is intended for use in general clinical practice in both the primary and secondary care settings. Management of acute GI bleeding currently requires an interdisciplinary approach, which may involve general practitioners, emergency and internal medicine physicians, gastroenterologists, hepatologists, endoscopists, surgeons, critical care practitioners, hematologists, biopathologists, intensive care specialists, nurses, and interventional radiologists, depending on local practice.

## Treatment of anemia

### Preliminary considerations

Acute GI bleeding results in blood loss, which, in extreme cases, can lead to hypovolemic shock and death. Although several international consensus/guidelines were published between 2012 and 2021 by the American Society for Gastrointestinal Endoscopy, the European Society of Gastrointestinal Endoscopy, and the American College of Gastroenterology (27, 51–55), the importance of correcting anemia and iron deficiency often remains undervalued or underestimated within the complexities of managing GI bleeding.

Until recently, RBC transfusion was considered a key treatment for acute blood loss anemia for many patients. However, its use in this setting is subject to substantial variability, with observational studies suggesting that RBC transfusion is used in 25–43% of cases of GI bleeding overall and that over 40% and 60% of transfusions in patients with upper and lower GI bleeding, respectively, are given in the absence of clinically significant anemia (hemoglobin ≥ 8 g/dL) (5, 56–58). More recently, goal-directed fluid therapy with restrictive volume restitution (59–61) has started to be used to correct hemodynamic instability, achieve hemodynamic control, and avoid hypovolemic shock, with RBC transfusions only recommended for patients with symptomatic anemia who do not respond to fluid therapy. Adaptation of transfusion criteria to align with clinical practice guidelines and expert reviews (26, 29, 57, 62–65) is therefore required to help optimize the use of transfusion resources and improve patient outcomes and clinical results.

Before determining what, when, and how to transfuse, certain considerations must be highlighted. First, it should be noted that hemoglobin levels are not always indicative of the full extent of blood loss in patients with GI bleeding. For example, decreased hemoglobin levels can be caused by fluid movement from the interstitial space to the vascular compartment, and infusion of intravenous fluids or overhydration may also lead to false low-concentration readings. A “normal” initial hemoglobin level, therefore, does not exclude the possibility of severe bleeding. Hemodynamic parameters may provide a better overview of the extent of blood loss together with regular monitoring of hemoglobin levels in the event of potentially severely bleeding lesions.

Second, excessive blood volume replacement can lead to an increased risk of rebleeding. This is because vasodilation and the subsequent increase in blood pressure can result in erosion of newly formed hemostatic plugs and dilution of clotting factors. Furthermore, the impact of hypothermia on *in vivo* coagulation must be considered. Overexpansion of plasma volume in patients bleeding because of portal hypertension may also induce an increase in portal pressure that may favor recurrent bleeding. A restrictive transfusion model that precludes the use of blood to increase blood volume is therefore recommended (29, 57), and RBC units should be transfused 1 unit at a time (66, 67), except in severe cases or in the case of uncontrolled active bleeding.

Third, the source of bleeding should be identified and the management of clotting disorders in patients with GI bleeding should also be considered. Thrombocytopenia is uncommon and is observed in only 5% of patients with upper GI bleeding and 1% of those with lower GI bleeding (68, 69). International normalized ratio (INR) levels > 1.5 are detected in 15% and 11% of patients with upper and lower GI bleeding, respectively, due primarily to the presence of liver failure or the use of oral anticoagulants such as warfarin or acenocoumarol (7, 68). Platelet transfusions are recommended for patients who have significant bleeding and a platelet count < 30 × 10^9^/L (66), particularly in cases of acute bleeding related to portal hypertension. It is not recommended to correct slight coagulation abnormalities with fresh frozen plasma transfusions in patients who have been receiving vitamin K antagonist treatment (66). The use of prothrombin complex concentrates and vitamin K to reverse the effects of oral anticoagulants is usually preferred.

Finally, consideration should be given to the adverse consequences that can accompany transfusion (Table 1) and the associated costs [e.g., in one European analysis, the estimated cost of a 2-unit RBC transfusion was €878 (70)].

**TABLE 1:** Acute and delayed adverse reactions associated with blood transfusion (64, 81).

Reaction type	Acute transfusion reactions (incidence)	Delayed transfusion reactions (incidence)
Immunologic	Acute hemolytic transfusion reaction (1/6,000)	Delayed hemolytic transfusion reaction
	Febrile non-hemolytic transfusion reaction (1/300)	Alloimmunization against cell antigens (also against platelets and leukocytes) (1/5–100)
	Cutaneous allergic transfusion reaction and urticaria (1/50–100)	Graft-versus-host disease
	Anaphylactic reaction (1/20,000–50,000)	Transfusion-related immunomodulation
	Acute non-cardiogenic pulmonary edema: transfusion-related acute lung injury (1/1,000–5,000)	Post-transfusion purpura
	Fatal hemolysis (1/1,000,000)	
	Transfusion-associated immunomodulation	
Non-immunologic	Bacterial contamination (1/5,000,000)	Transfusion-transmitted infections*: virus (e.g., HAV/HBV/HCV/HEV, HIV 1-2, West Nile, HTLV I-II, cytomegalovirus, *Herpesviridae*, TTV, SEN-1, SARS), protozoa (e.g., malaria, babesiosis, Chagas disease), prion (new variant of Creutzfeldt-Jakob disease)
	Transfusion-associated circulatory overload (1/100–500)	Transfusional hemosiderosis (iron overload)
	Transfusion-related acute lung injury (1/1,000–5,000)	
	Hypotension	
	Non-immunologic hemolysis	
	Others: hypocalcemia, hyperkalemia (cardiac arrest), hypothermia, hyperglycemia, etc.	

HAV/HBV/HCV/HEV, hepatitis A/B/C/E virus; HIV, human immunodeficiency virus; HTLV, human T-lymphotropic virus; SARS, severe acute respiratory syndrome; TTV, Torque teno virus.

*Malaria 1/4,000,000; HIV < 1/2,000,000; HCV < 1/1,000,000; HTLV: 1/641,000; HBV: 1/100,000.

### Management of anemia due to acute gastrointestinal blood loss

**FIGURE 1:**
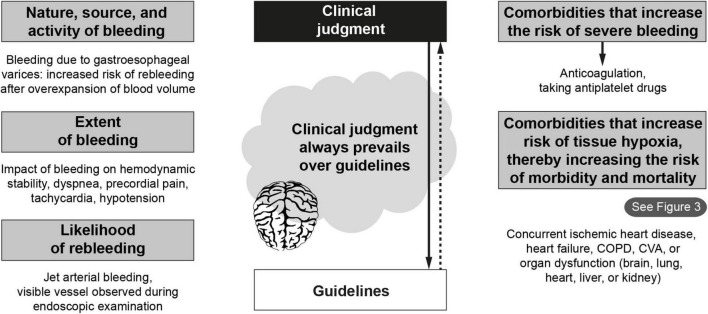
Factors influencing the decision to give transfusions. COPD, chronic obstructive pulmonary disease; CVA, cerebrovascular accident.

The GI bleeding protocol presented here should be integrated into a hospital’s wider PBM program. Many factors should be considered when deciding whether to give an RBC transfusion to patients with anemia. The criteria used in PBM to decide what, who, and when to transfuse are broadly based on the severity of the bleeding, the impact on hemodynamic stability, the source and activity of the bleeding, the likelihood of rebleeding, and the presence of comorbidities (71) that may affect bleeding control or increase the risk of tissue hypoxia (Figure 1). Blood transfusion is an early example of “personalized medicine” as treatment decisions should be individualized and guidelines should not supersede the clinical judgment of the treating physician when deciding which patients should undergo transfusion. Adequate replenishment of blood volume is essential to maintain and optimize organ perfusion. However, this should generally involve crystalloid- and colloid-based fluid therapy rather than blood transfusion. The following section includes some important considerations in the management of patients with anemia due to acute blood loss.

1. Patients receiving anticoagulant treatment are at increased risk of severe or persistent bleeding; therefore, this should firstly be reversed. In contrast, antiplatelet drugs should only be discontinued if absolutely necessary (6, 62).
2. Dyspnea, chest pain, tachycardia, hypotension that is refractory to initial blood volume replacement, obnubilation, and oliguria are warning signs or symptoms of acute blood loss (27).
3. Active bleeding or identification of a visible vessel during endoscopy indicates an increased risk of rebleeding (27).
4. The presence of gastroesophageal varices also increases the risk of rebleeding if the plasma volume is overexpanded following transfusion (19, 21, 29).
5. Factors that increase the risk of a vascular event (e.g., history of coronary artery disease, arrhythmias, and/or heart failure – see section below) add to the complexity of the decision-making process (55).

### Clinical evaluations used within the patient blood management protocol

It is necessary to consider and record any variables that may influence the decision whether to administer a RBC transfusion, as well as variables that have an impact on the rate of transfusion or route of administration of iron replacement therapy (oral vs. intravenous) (66). This includes:

• The patient’s medical history, especially any recent history of ischemic or thrombotic events, cardiopulmonary disease, chronic kidney disease, imminent surgery (within < 30 days), severity of bleeding and associated anemia, or any clinical condition that may interfere with oral iron availability or absorption (e.g., iron tolerance or refractivity).
• As a minimum, the following laboratory tests that could indicate the presence or absence of iron deficiency should also be performed: hemoglobin level; numbers of RBC and reticulocytes; RBC distribution width; mean corpuscular hemoglobin; mean corpuscular volume; serum ferritin and transferrin saturation (TSAT) levels; and measurement of C-reactive protein, creatinine, and urea levels.
• Additional complementary tests may be performed depending on the clinical scenario, such as more extensive laboratory tests including the measurement of serum vitamin B_12_, folate, haptoglobin, soluble transferrin receptor, or lactate dehydrogenase levels, and reticulocyte hemoglobin content.
• A GI endoscopy or computed tomography angiogram may also be performed to identify the cause and extent of the bleeding as well as the presence of endoscopic warning signs of rebleeding (according to the Forrest Classification System), which could reduce the risk of rebleeding and transfusion. Both endoscopy and angiography can offer additional and effective therapeutic resources in the control and cessation of bleeding.

### Restrictive red blood cell transfusion within patient blood management

Various measures for avoiding transfusion are proposed by the PBM strategy. These include testing for and evaluating any anemia (and its origin) or coagulopathy issues, and providing specific treatment (e.g., intravenous iron replacement therapy in the case of iron deficiency anemia, reversal of anticoagulant/antiplatelet therapy, prohemostatic drugs). Strategies to reduce the risk of bleeding associated with invasive procedures should also be employed. Iatrogenic blood loss due to the taking of excessive analytical samples should be avoided by using pediatric tubes and non-invasive point-of-care monitoring for hemoglobin levels, INR, hypoxia, etc. Moreover, the minimum clinically effective transfusion volume should be used (where feasible) (72).

### Transfusion thresholds

**FIGURE 2:**
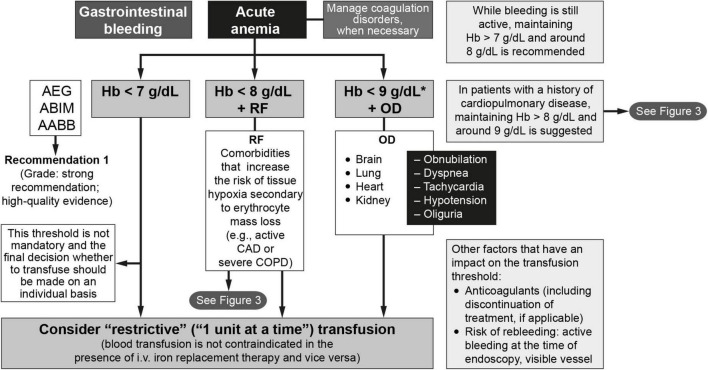
The management of anemia and iron deficiency in patients with acute gastrointestinal bleeding (64, 81). AABB, American Association of Blood Banks; ABIM, American Board of Internal Medicine; AEG, Asociación Española de Gastroenterología; CAD, coronary artery disease; COPD, chronic obstructive pulmonary disease; CVA, cerebrovascular accident; Hb, hemoglobin; i.v., intravenous; OD, organ dysfunction; RF, risk factors. * < 10 g/dL if severe bleeding.

**FIGURE 3:**
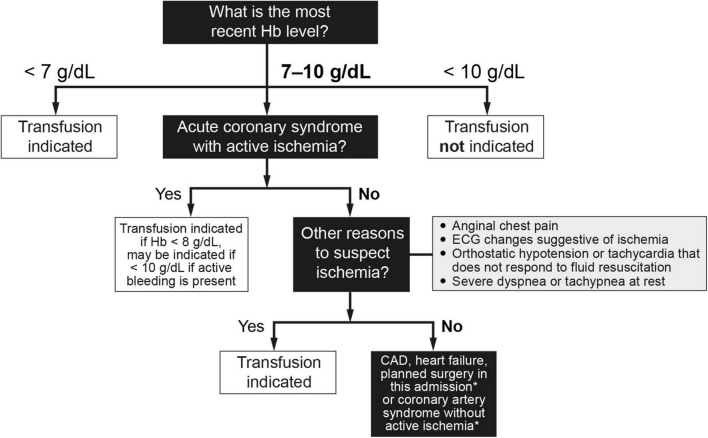
Indications for red blood cell transfusion in the presence of cardiovascular risk factors (64, 77–81). In cases of both acute hemorrhage and chronic blood loss, the decision to transfuse red blood cell concentrates does not exempt the indication to replenish iron stores, because 1 red blood cell unit only provides 200 mg of iron. This consideration is especially important in patients with CAD and/or heart failure. CAD, coronary artery disease; ECG, electrocardiographic; Hb, hemoglobin. *Consider transfusion if Hb < 7.5 g/dL.

An algorithm depicting the indications for RBC transfusion in patients with acute GI bleeding is shown in Figure 2. The protocol establishes the following guidelines:

1. In the absence of risk factors and warning signs or organ dysfunction, a hemoglobin level of < 7 g/dL is often used as the cutoff for transfusion when a restrictive approach is being used (16, 17, 20, 73, 74). This threshold is not mandatory and the final decision whether to transfuse should be made on an individual basis. Close monitoring alone without transfusion is an option for patients with hemoglobin levels below this threshold who have no comorbidities or symptoms, are hemodynamically stable, have inactive bleeding, and have a low risk of rebleeding.
2. The question of when to transfuse the patient who has ischemic heart disease and/or heart failure remains controversial. In fact, taken together, the short- and long-term findings from available clinical trials indicate that the question of non-inferiority and/or superiority of the two transfusion approaches (restrictive vs. liberal) in these patients remains unanswered (25, 75–81). The greatest difficulty lies in the large number of possible scenarios. An algorithm to determine if transfusion is indicated in patients with cardiovascular risk can be found in Figure 3 (81). For most patients in this category, the recommendation is to transfuse to maintain hemoglobin levels > 8 g/dL but no more than 9 g/dL. Therefore, following the recommendations of the American Association of Blood Banks (64) and based on the clinical trial results available to date, restrictive RBC transfusion should be considered for patients with a hemoglobin level of < 8 g/dL in the following cases (25, 75–81):

• Patients with an acute coronary syndrome (e.g., acute myocardial infarction or with known coronary artery disease and unstable angina) and who have ongoing ischemia despite anti-ischemic therapy, such as medical therapy or angioplasty. Lower target hemoglobin levels are often used for patients whose signs and/or symptoms of ischemia resolve with anti-ischemic therapy.
• Patients with other findings suggestive of active ischemia, including anginal chest pain, electrocardiographic changes suggestive of ischemia, orthostatic hypotension or tachycardia that does not respond to fluid resuscitation, or severe dyspnea or tachypnea at rest. Clinical judgment is required to determine if a patient’s symptoms signify active ongoing ischemia (i.e., transfusion is indicated) or merely reduced oxygen carrying capacity (which may be treated without transfusion). Signs or symptoms that do not necessarily warrant transfusion include irritability, weakness, tiredness, or exertional dyspnea. Conversely, these signs/symptoms may not always be present in a patient with ischemia (e.g., a patient receiving a beta-blocker may not have tachycardia).
• Patients with chronic obstructive pulmonary disease (COPD).
• The presence of vascular risk factors for arteriosclerosis (e.g., diabetes, hypertension, dyslipidemia, smoking) alone are not sufficient to warrant transfusion, unless the patient has signs of active bleeding and/or hemodynamic instability that is not corrected with fluid replacement.
• The threshold for patients with a history of heart failure is less clear, but a hemoglobin threshold of between 7 and 8 g/dL (70–80 g/L) is likely to be appropriate for most patients. Hospitalized patients with heart failure are especially challenging to manage, and the improvement in oxygenation from transfusion must be balanced against the risks of worsening heart failure due to the volume of transfused blood. When RBC transfusion is required in a patient with heart failure, careful attention to volume status is recommended, including adjustment of transfusion rate and use of supplemental diuretics as needed to avoid volume overload.
3. RBC transfusion should only be considered for patients with hemoglobin levels > 8 g/dL but < 9 g/dL in the presence of organ (heart, brain, lung, or liver) dysfunction. In this situation, clinicians should consider the advantages and disadvantages of use/overuse of transfusion, regardless of any laboratory test results. The volume of blood transfused should not exceed the amount required to relieve symptoms of anemia or to achieve a hemoglobin level of 7–8 g/dL (which is also considered safe for stable, non-cardiac patients). An important point to consider is that RBC transfusion does not exempt the need for intravenous iron replacement therapy, since 1 RBC unit provides no more than 200 mg of iron.

### Management of chronic anemia due to occult blood loss

**FIGURE 4:**
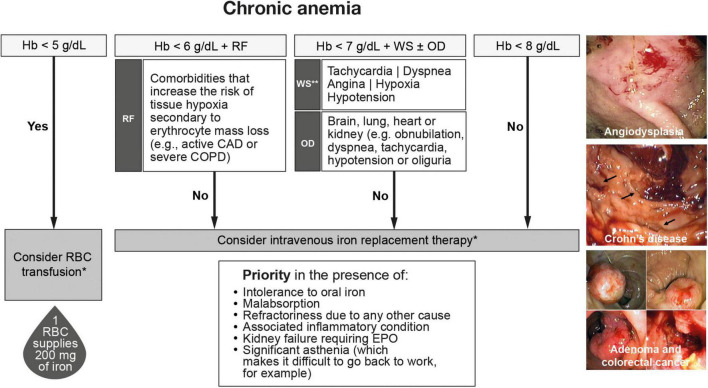
Algorithm for the management of chronic anemia associated with gastrointestinal blood loss. CAD, coronary artery disease; COPD, chronic obstructive pulmonary disease; EPO, erythropoietin; Hb, hemoglobin; i.v., intravenous; OD, organ dysfunction; RBC, red blood cell; RF, risk factors; WS, warning signs. *RBC transfusion is not enough to replenish iron stores; 1 RBC unit supplies 200 mg of iron; **Warning signs in patients with Hb levels < 7 g/dL could be triggered by events such as atrial fibrillation, sepsis with a systemic inflammatory response, or an “acute on chronic” bleeding episode.

Hemoglobin thresholds for transfusion in the case of chronic anemia associated with occult GI bleeding (Figure 4) differ from those used in the setting of acute bleeding (Figure 2) (73, 74, 82). RBC transfusion should be considered for patients with hemoglobin levels < 5 g/dL and for those with hemoglobin levels < 6 g/dL in the presence of risk factors such as cardiopulmonary failure, ischemic heart disease, cardiac arrhythmia, and COPD. These hemoglobin thresholds are supported by studies showing how in patients with severe chronic anemia (hemoglobin < 6 g/dL), and even extreme anemia (hemoglobin ≤ 5 g/dL) due to digestive or gynecological blood loss, third-generation intravenous iron is effective and safe for the rapid correction of anemia (29, 73). This policy helps to avoid unnecessary blood transfusions. Transfusion should also be considered for patients with hemoglobin levels < 7 g/dL with associated warning signs and symptoms of organ dysfunction, such as dyspnea, precordial pain, tachycardia, hypoxia, or orthostatic hypotension. Patients with hemoglobin levels > 7 g/dL and no symptoms of organ dysfunction should undergo observation and correction of any iron deficiency. As mentioned previously, if a patient receives a transfusion, this does not exempt the need for intravenous iron replacement therapy, and vice versa. RBC transfusions should not be administered to patients who only have iron deficiency, except in those who are hemodynamically unstable.

### Treatment of iron deficiency

#### Preliminary considerations

Iron is crucial for erythropoiesis and oxygen transport, and it also serves important roles in cell energy production, maturation of the immune system, and efficient organ function (83–86). Although iron deficiency anemia is the most prevalent cause of anemia worldwide, not all anemias are due to an iron deficit. Furthermore, many patients have been found to have iron deficiency who do not have anemia. The criteria used to diagnose iron deficiency are summarized in Table 2. It should be noted that the terms *iron deficiency* and *iron deficiency anemia* are often confused in publications and these terms are not necessarily synonymous (87–89). GI-related issues that may lead to the onset of iron deficiency and iron deficiency anemia are shown in Table 3.

**TABLE 2:** Criteria and other indicators for diagnosing iron deficiency.

Criteria for diagnosing iron deficiency: • Ferritin (acute-phase reactant) < 30–100 μg/L* • Transferrin > 300–350 mg/dL^†^ • Transferrin saturation < 20%^‡^
Markers of iron deficiency in circulating red blood cells: • Mean corpuscular volume < 81 fL • Mean corpuscular hemoglobin < 28 pg/cell • Red blood cell distribution width > 15% • Hypochromic red blood cells > 5%
Other indicators of iron deficiency: • sTfR > 2.0 mg/L • sTfR/log ferritin index > 2.0 • Mean reticulocyte hemoglobin content < 27.2 pg/cell • Low hemoglobin density > 5–10%

sTfR, soluble transferrin receptor.

*Since ferritin is an acute-phase reactant, levels < 100 μg/L may be indicative of iron deficiency in the presence of inflammation.

^†^In iron deficiency due to acute gastrointestinal bleeding, transferrin levels are often relatively low (200–250 mg/dL) because of associated protein loss.

^‡^In iron deficiency due to acute gastrointestinal bleeding, transferrin saturation is often relatively high (≥ 45%) because of associated low transferrin levels.

**TABLE 3:** Gastrointestinal causes of iron deficiency and iron deficiency anemia.

Factors associated with gastrointestinal bleeding (macro or microscopic): • Gastroduodenal peptic ulcer/*Heliobacter pylori* infection • Hemorrhage due to esophagogastric varices • Portal hypertension gastropathy • Gastric antral vascular ectasia • Angiodysplasia • Inflammatory bowel disease • Use of acetylsalicylic acid or other non-steroidal anti-inflammatory drugs • Gastrointestinal malignancies • Diverticular bleeding • Hemorrhoid bleeding • Postoperative bleeding • Large hiatus hernias (Cameron lesions) • Parasitic infections
Factors associated with impaired absorption due to limited availability of or damage to enterocytes: • Low-iron diets • Chronic autoimmune atrophic gastritis • Celiac disease/non-celiac gluten sensitivity • Inflammatory bowel disease • Intestinal lymphoma • Bariatric surgery/gastric bypass surgery • Short bowel syndrome • Gastrectomy or gastrojejunostomy • Bacterial overgrowth

Acute GI bleeding leads to a reduction in RBC volume resulting in a need for RBC regeneration, which subsequently can lead to iron deficiency and iron deficiency anemia; hence, it is important to consider the use of iron replacement therapy in these patients. Although the anemia in acute GI bleeding is initially due to blood loss, if iron stores are reduced or iron absorption is disrupted, the iron deficit will continue to be observed over time. For example, > 60% of patients enrolled in a Spanish study developed iron deficiency anemia within 1 month of non-variceal upper GI bleeding (90). Risk factors for the development of iron deficiency anemia were age > 75 years, initial ferritinemia < 65 μg/L, initial hemoglobin levels < 10 g/dL, and TSAT < 10% at day 5. After an episode of upper GI bleeding, blood urea levels temporarily rise due to the absorption of extravasated urea nitrogen in the intestinal lumen, and urea levels may therefore be higher than creatinine levels. Consequently, blood urea levels > 80 mg/dL at admission may indicate significant bleeding and an increased risk of anemia. The use of iron replacement therapy in patients with chronic iron deficiency anemia can lead to a reduction in RBC transfusions (91) and a consequent reduction in the associated risks. Iron replacement therapy should be started as soon as iron deficiency is detected, to replenish iron stores and restore normal erythropoiesis as soon as possible (92, 93).

#### Advantages and limitations of oral and intravenous iron administration

The choice between oral and intravenous iron replacement therapy depends on various factors, such as the severity and speed of the onset of anemia, cost, the availability of existing formulations, patient tolerance to oral iron, patient preferences, and the existence of other limiting factors (e.g., malabsorption of oral iron, inflammation, or allergies to intravenous iron) (86, 94–96). Under suitable conditions, oral iron is effective, readily available, inexpensive, and well tolerated (97–99). For example, many patients who have mild iron deficiency anemia associated with chronic fecal occult blood loss may be effectively managed with oral iron. However, treatment with oral iron (especially iron sulfate) is associated with GI side effects in many patients (100). Other limitations to oral iron therapy are low levels of adherence, lack of suitability for patients with severe bleeding or continuous occult blood loss, the excessive time (months) required to replenish iron stores, and the lack of clarity regarding total costs, which may be higher than initially expected when absenteeism/presenteeism considerations are also taken into consideration. Newer intravenous iron compounds are considered safer than blood transfusion, have fewer GI side effects than oral iron (100, 101), and are also associated with improved/guaranteed adherence (67, 91, 94, 101, 102). These compounds also provide the total iron dose required (often in a single infusion), quickly increase hemoglobin levels, and promote more effective replenishment of iron stores in comparison with oral iron (67, 91, 94, 101–104). Much of the data supporting the efficacy and safety of intravenous iron in patients with GI bleeding come from studies of ferric carboxymaltose (FCM) (33, 91, 101, 102). A prospective study showed that 1,500/2,000 mg of intravenous FCM (given over 2 infusions) increased hemoglobin and iron levels faster and more effectively when compared with 6 weeks of oral ferrous sulfate treatment in patients with anemia due to acute GI bleeding (102). FCM was also better tolerated than oral iron and associated with significantly improved quality of life (102). Furthermore, a retrospective study showed that a single 1,000-mg FCM infusion effectively increased hemoglobin levels in elderly patients who had comorbidities and acute GI bleeding (including some who were hemodynamically unstable), thereby supporting the use of a restrictive transfusion policy (33). Hemoglobin levels were also increased, and transfusion rates significantly reduced in a retrospective study of FCM in patients with chronic GI bleeding who had previously been receiving chronic transfusion support (91). Similar effects on iron parameters have been reported for other intravenous iron formulations (105–107).

There are, however, limitations to the use of intravenous iron. These include the requirement for monitoring during infusion, the risk of infusion-related reactions (although these are extremely uncommon), the requirement for equipment and staff training, and the potential for increased costs (67, 91, 94, 101, 102). Furthermore, although increases in hemoglobin levels occur more quickly than with oral iron, it may still take 3–5 days for levels to improve following infusion. Therefore, blood transfusion is a necessary therapeutic option to maintain the transport of oxygen to the tissues in cases of severe anemia where alternative treatments are not available or where it is not possible to wait for these to take effect. Nonetheless, intravenous iron can be complementary to blood transfusion to treat any underlying iron deficiency (i.e., blood transfusion does not contraindicate intravenous iron treatment or vice versa). The decision about when intravenous iron should be administered (before, during, or after blood transfusion) is related to the patient’s hemodynamic status. In patients with hemodynamic instability, alarm signs, or organ dysfunction, transfusion should be prioritized, and transfusion should not coincide with the intravenous iron infusion, i.e, it should not be administered in the same line nor at the same time. Intravenous iron should be administered once the transfusion of the first unit has been completed, after clinical and analytical evaluation, and in the absence of any adverse reactions. However, in the event of hemodynamic stability, with stabilized and controlled bleeding, and in the absence of clinical signs or symptoms suggesting that an urgent transfusion is required, intravenous iron can be administered (over 15–30 min), while the pre-transfusion compatibility tests are being performed by the transfusion service and the red blood cell concentrates are awaited. In general, we must aim to transfuse the minimum number of blood units possible to achieve the required clinical effect alongside treatment specific to the cause of the anemia. Adverse effects associated with intravenous iron infusion can include nausea, headache, dizziness, hypertension, skin rash, injection-site reactions, hypophosphatemia, increased alanine aminotransferase levels, and (extremely rarely) hypersensitivity reactions. In general, the third-generation compounds have the safest profiles. These agents are better tolerated and are associated with a lower risk of infusion-related/anaphylactoid reactions, and a reduced generation of non-transferrin bound free iron when compared with earlier compounds (108, 109). However, each intravenous iron product is unique and thus it is not possible to extrapolate data from one product to compare with that of another (110). The use of FCM also permits a higher iron dose to be given over fewer, shorter infusions (only 1 or 2 doses of 1,000 mg, each given over a 15-min infusion, are usually required in comparison with, for example, multiple, longer infusions for iron sucrose) (103, 109, 111, 112).

#### Indications for the use of intravenous iron

The indications for the use of intravenous iron in patients with GI bleeding are shown in Table 4. In patients with acute GI bleeding and uncontrolled hypertension or hemodynamic instability (as indicated by a systolic blood pressure < 90 mmHg or heart rate > 100 bpm), blood volume should initially be replenished with fluids and transfusion is not needed. However, in the case of acute anemia due to severe GI bleeding, RBC transfusion is recommended.

**TABLE 4:** Indications for the use of intravenous iron in patients with gastrointestinal bleeding.

While in hospital	After hospital discharge
Where there is a need for rapid correction of moderate/severe iron deficiency anemia • Where there is iron deficiency and concomitant inflammatory status (CRP > 5 mg/dL) causing reduced absorption of oral iron due to the effects of hepcidin on ferroportin • In patients with gastrointestinal bleeding who also meet any of the following criteria: ° Need for imminent surgery (<30 days) with estimated perioperative blood loss > 1–1.5 L* ° Need for invasive surgery with a risk of significant bleeding ° Need for erythropoiesis-stimulating agent treatment (preemptive intravenous iron is given to prevent non-response to EPO – the primary cause of which is functional iron deficiency^†^) • Need for artificial feeding (parenteral or enteral) • As an alternative to blood transfusion (e.g., in patients who reject blood transfusion based on religious grounds or personal beliefs)	Where there is a need for rapid correction of moderate/severe iron deficiency anemia • When iron deposits are very low and there is a need for rapid repletion to initiate erythropoiesis • In the event of oral iron therapy failure due to: ° Intolerance of side effects that do not respond to recommended measures for improving tolerability ° Poor adherence ° Monthly increments of < 1 g/dL of hemoglobin (iron deficiency anemia that does not respond to oral iron)^‡,§^ • Where there is a contraindication to oral iron preparations or another reason why oral iron cannot be used

CRP, C-reactive protein; EPO, erythropoietin.

*For example, when bleeding is due to a resectable malignancy or the patient is admitted while awaiting orthopedic hip surgery.

^†^A situation in which iron requirements exceed available iron stores. This term implies iron status with ferritin < 100 μg/dL and a transferrin saturation < 20% (or ferritin < 500 μg/L and a transferrin saturation < 30% in the presence of chronic kidney failure).

^‡^Intravenous iron replacement therapy should be considered prior to discharge when factors that limit absorption have been identified during hospitalization.

^§^Reasons that may explain refractoriness to oral iron include: interference with absorption (hypoacidity secondary to chronic autoimmune atrophic gastritis or the use of proton pump inhibitors, lymphocytic duodenosis due to Helicobacter pylori infection); reduced surface area available for absorption (gastrectomy, bariatric surgery); gluten-sensitive enteropathy or other clinical conditions that cause malabsorption, including edematous bowel loops due to heart or chronic kidney disease or severe hypoalbuminemia; or active inflammatory bowel disease. Other inflammatory conditions, such as systolic heart failure and left ventricular ejection fraction < 45%, should also be considered.

None of the scenarios that support the use of RBC transfusion preclude the use of intravenous iron for the treatment of any associated iron deficiency. It should be remembered that 1 unit of RBC concentrate provides approximately 200 mg of iron and, therefore, is not enough to replenish iron stores (estimated at up to 2 g in patients with hemoglobin levels < 10 g/dL post-bleeding and a body weight of 75 kg; Table 5) (111, 113).

**TABLE 5:** A simplified formula for calculating the required dose of ferric carboxymaltose, based on patient body weight and hemoglobin levels* (110, 112).

	Ferric carboxymaltose dose (mg)	
	Weight 35 to < 70 kg	Weight ≥ 70 kg
Hemoglobin level, g/dL		
<10	1,500	2,000
10 to < 14	1,000	1,500
≥14	500	500

*The maximum recommended cumulative dose of ferric carboxymaltose is 1,000 mg/week.

In patients with iron deficiency/iron deficiency anemia due to chronic GI bleeding, treatment with intravenous iron can be a very good alternative to RBC transfusion, thereby reducing or avoiding the need for transfusions (73). However, transfusion should be used in these patients when pharmacologic treatment of anemia has failed or in cases of severe anemia.

Contraindications to intravenous iron include persistent bacteremia, serious known allergy, hypersensitivity to other parenteral iron-containing products, TSAT > 45% (or ferritin levels > 500 μg/L with TSAT > 25% in patients with inflammatory conditions), or hemochromatosis/hemosiderosis/porphyria cutanea tarda. A history of bronchial asthma or severe eczema also increases the risk of a hypersensitivity reaction after administration of intravenous iron, and this aspect should be considered before establishing the need for intravenous iron treatment in such patients. Intravenous iron should also generally not be used during the first trimester of pregnancy.

Until recently, intravenous doses of iron were often calculated using the Ganzoni formula (114). This formula is based on body weight, actual compared with target hemoglobin levels, and iron stores. A limitation of this formula is that it underestimates the dose of iron required in patients with acute bleeding and is only reliable in patients with pure iron deficiency. In patients with mixed anemia (where anemia is partly due to other causes), use of the Ganzoni formula may lead to excessive iron replenishment and subsequent iron overload. A simple and efficient method for calculating the dose of intravenous iron based on body weight and hemoglobin levels has been developed based on information included in the summary of product characteristics for FCM (Table 5) (111, 113). This is commonly used in hospital settings in the context of GI bleeding, especially in severe cases. In a randomized, placebo-controlled clinical trial of oral vs. intravenous iron in patients who had experienced an upper gastrointestinal hemorrhage, the authors suggested that treatment with intravenous FCM, even when given at lower than standard doses, was significantly beneficial compared with oral ferrous sulphate in patients with lower body weight (115), although this finding needs to be validated in studies involving a larger number of patients.

## Summary

GI bleeding is common in hospital settings and requires management by an interdisciplinary team (3). Blood volume depletion, loss of RBC mass, and associated clotting disorders require support and replacement strategies. Although transfusions undoubtedly save lives and are a fundamental pillar of the management of severe GI bleeding, they remain one of the most overused medical procedures and are associated with many “Do Not Do Recommendations” (66). The decision to give an RBC transfusion is challenging and should take into account individual patient characteristics, together with the source, activity, and extent of bleeding and the patient’s clinical tolerance of anemia. Several studies support the use of clinical decision-making tools to promote PBM and restrictive transfusion practices, and to improve RBC utilization, even in high-risk patients (33, 50, 74, 91, 116–118). In some patient groups, a restrictive strategy can reduce unnecessary use of allogeneic transfusions and is associated with equivalent or better outcomes than a liberal strategy.

The decision to administer oral or intravenous iron to a patient with bleeding depends on multiple factors, including the severity of anemia, the presence of inflammation, whether a rapid increase in hemoglobin levels and replenishment of iron stores are needed to benefit patient symptoms and quality of life, costs, and patient adherence to treatment with oral iron (94, 97, 103, 119, 120). The protocol presented here is the result of a thorough review performed by gastroenterologists, hepatologists, hematologists, and PBM experts with solid training in the implementation of PBM-based policies. It also reflects current clinical practice in Spain regarding the management of anemia and iron deficiency in patients with acute or chronic GI bleeding. The protocol has been designed to maintain a balance between the use and overuse of transfusion and advises on the safe use of oral versus intravenous iron in these settings. This has permitted the introduction of a prospective database – AEG-REDCap (Research Electronic Data Capture Service of the AEG) – in many Spanish hospitals, in which the clinical characteristics of patients who are admitted because of GI bleeding can be recorded. Having a prospective registry of cases should help avoid the confounding bias that may result from inadequate variability in clinical practice.

In summary, collaboration between physicians in direct contact with patients who have GI bleeding and hematologists and other specialists involved in PBM is important and should contribute to the improved future management and correction of iron deficiency anemia. While blood is a costly resource, which is dependent on the participation of donors, replenishment of iron stores in patients contributes to an improvement in health-related quality of life and an overall reduction in healthcare costs.

## Author contributions

MM and JAGE developed the concept for the manuscript and wrote the main body of the article. All authors contributed to manuscript revision, read, and approved the submitted version.
